# The Role of Self-Compassion and Shame-Proneness Among Anorexia and Bulimia Nervosa

**DOI:** 10.3390/healthcare14010047

**Published:** 2025-12-24

**Authors:** Lorenzo Antichi, Chiara Rossi, Elisa Scerrati, Daniel Kořínek, Jan Benda, Giuseppe Riva

**Affiliations:** 1 Department of Psychology, Università Cattolica del Sacro Cuore, Largo Gemelli 1, 20123 Milan, Italy; lorenzo.antichi@unicatt.it (L.A.); elisa.scerrati@unicatt.it (E.S.); giuseppe.riva@unicatt.it (G.R.); 2 Department of Psychology and Health Sciences, Università Telematica Pegaso, 80132 Naples, Italy; 3 Psychotherapeutic and Psychosomatic Clinic ESET, 100 00 Prague, Czech Republic; daniel.korinek@centrum.cz; 4 Department of Psychiatry, First Faculty of Medicine, Charles University, 121 08 Prague, Czech Republic; 5 Department of Psychology, Faculty of Arts, Charles University, 116 38 Prague, Czech Republic; psychoterapeut@gmail.com; 6 Applied Technology for Neuro-Psychology Laboratory, Istituto di Ricovero e Cura a Carattere scientifico, Istituto Auxologico Italiano, 20145 Milan, Italy

**Keywords:** eating disorders, self-compassion, shame-proneness, latent profile analysis, anorexia nervosa, bulimia nervosa

## Abstract

**Background**: Self-compassion (SC) and shame-proneness (SP) are likely transdiagnostic factors implicated in the onset and maintenance of eating disorders (EDs). However, limited research has examined how these variables vary across distinct ED symptom profiles. To address this gap, this exploratory study aimed to identify latent symptom profiles among individuals diagnosed with Anorexia Nervosa (AN) or Bulimia Nervosa (BN) and assess whether SC and SP levels and their association differ across classes. **Methods**: A clinical sample of 55 women with AN or BN completed self-report measures for assessing drive for thinness (DT), bulimia (BUL), body dissatisfaction (BD), self-compassion (SC), and SP. Latent Profile Analysis (LPA) was conducted, followed by ANOVA and moderation analysis. **Results**: LPA revealed three distinct profiles: (1) Low-symptom (i.e., low DT and BUL, moderate BD), (2) Restrictive (i.e., high DT and BD, low BUL), and (3) Multi-symptomatic (i.e., medium-high DT, BUL, and BD). SC significantly differed across profiles, with the Low-symptom group reporting higher SC than the others. No significant differences in SP were found. SC was negatively associated with ED symptoms and significantly moderated the relationship between SP and BD, but not DT or BUL. **Conclusions**: Findings highlight the heterogeneity of ED symptomatology and the importance of SC as a protective factor, particularly against body dissatisfaction.

## 1. Introduction

Anorexia Nervosa (AN) and Bulimia Nervosa (BN) are two of the most common eating disorders (EDs). However, little is still known about their etiopathogenetic processes. Over the years, psychopathology research has investigated the etiology of BN and AN. In addition to biological factors such as heredity and serotonin-related appetite regulation, social (e.g., exposure to the thinness ideal), developmental (e.g., trauma, sexual abuse), and psychological (e.g., impulsivity, antagonism, perfectionism) factors have been proposed [1,2,3]. Contemporary research increasingly emphasizes the importance of identifying transdiagnostic mechanisms that may contribute to both the development and persistence of EDs, particularly given their frequent comorbidity with mood, anxiety, or obsessive-compulsive symptoms [4]. Among these, two factors gaining growing attention are self-compassion (SC) and shame-proneness (SP).

SC is conceptualized as the ability to face personal suffering with kindness, to recognize human imperfection, and to maintain emotional balance when committing personal mistakes or facing life challenges [5]. SC appears to be a protective factor for EDs [6], as higher SC is associated with reduced symptom severity, particularly restrictive eating behaviors and body dissatisfaction [7,8,9]. SC also seems to limit the internalization of sociocultural appearance ideals, fostering a more positive self-attitude and reducing vulnerability to body image disturbances [10]. Individuals with EDs often show low SC levels [11,12]. Thus, SC is increasingly considered an alternative resilience pathway, able to modulate negative emotional states, especially shame, implicated in ED maintenance [13].

Within this framework, SC is understood as a mindfulness-based way of relating to oneself, in which mindful awareness of suffering is a core component of the self-compassionate stance. To extend compassion to oneself, individuals must first notice painful thoughts and emotions with balanced, non-judgmental awareness; SC then adds an active attitude of kindness and recognition of common humanity [14]. This integration suggests that SC may function as a mindfulness-informed regulatory process, particularly relevant in EDs, where individuals tend to overidentify with perceived failures and appearance-focused self-schemas.

SP is defined as the tendency to experience a distressing emotional response resulting from negative global self-evaluation in the context of perceived transgressions or personal shortcomings [15]. In EDs, shame can manifest as internalized shame, external shame, or body-related shame, and it is a significant risk factor in both development and persistence [16,17]. SP is associated with restrictive dieting, binge eating, purging, and body dissatisfaction [18,19,20]. Shame may also reinforce social comparison, perfectionism, and internalization of unattainable appearance standards, amplifying feelings of inadequacy [21].

SC may buffer the detrimental effects of shame, particularly in relation to body dissatisfaction and self-disgust [11,22,23]. SC has been shown to moderate associations between shame-related memories and ED symptoms [11] and to weaken maladaptive cycles of self-criticism [7]. However, findings remain inconclusive, partly due to heterogeneous operationalizations of shame (e.g., global SP, body shame, external shame) and SC, as well as differences in clinical status, diagnosis, and socio-demographic characteristics [8].

Given the high degree of symptom heterogeneity and transdiagnostic presentations in EDs [24,25], it is crucial to examine whether SC and SP levels, and their interplay, differ across distinct ED symptom profiles. Such work can clarify how emotional processes contribute to specific clinical phenotypes and guide tailored interventions for high-risk individuals. Accordingly, although the present work was designed as an exploratory study, we formulated the following guiding research questions (RQ):

RQ1: Do distinct latent profiles of ED symptoms (i.e., drive for thinness, bulimic symptoms, and body dissatisfaction) emerge among women diagnosed with AN or BN?

RQ2: Do identified latent profiles differ in levels of SC and SP?

RQ3: Does SC moderate the association between SP and the three ED symptom dimensions?

## 2. Materials and Methods

The study received ethical approval from the Ethical Committee of the General University Hospital in Prague (Ref. No. 285/18 IS, D; study ID: 21/18 S-IV). Data were collected from two specialized clinical institutions: (a) The Center for Eating Disorders at the Department of Psychiatry, First Faculty of Medicine, Charles University and General University Hospital in Prague, where the diagnosis of an eating disorder was made by a physician upon admission as a condition for hospitalization (according to ICD-10); (b) ESET Psychotherapeutic and Psychosomatic Clinic, where patients entered outpatient psychotherapy under the care of the researcher based on a referral from their psychiatrist or general practitioner. In the latter setting, the ED diagnosis was confirmed through a clinical interview conducted by the fourth and fifth authors in accordance with ICD-10 guidelines. Once participants agreed to be enrolled in the study and signed the informed consent form, they completed measures assessing SC, SP, and ED symptom severity (i.e., DT, BUL, BD) and socio-demographic characteristics.

### 2.1. Sample

Overall, 55 female participants were recruited, with an average age of 26.18 years (SD = 9.10). Of these, 54.5% (N= 30) were diagnosed with AN (F50.0), and 45.5% (N = 25) with BN (F50.2). The majority of participants were single (i.e., 87.3%), did not have children (i.e., 81.8%), and held a tertiary education degree (i.e., 41.8%). The inclusion criteria were women aged 19–39 with a primary diagnosis of AN or BN, as defined by the International Classification of Diseases (ICD-10).

### 2.2. Instruments

Socio-Demographic Characteristics. Some socio-demographic information has been collected, including sex, marital status, number of children, education level, and age.

Eating Disorder Inventory [26,27]. Three EDI subscales were selected: DT measures dieting concerns, diet restrictions, and fear of weight gain; BUL assesses tendencies toward uncontrollable overeating, purging to lose weight, and feeling emotionally upset in response to these behaviors; BD assesses feelings related to the shape and size of one’s body. Respondents rated each statement on a six-point Likert-type scale (0 = never; 5 = always). Higher scores indicate greater symptom severity. Good reliability was found for all subscales of the Czech version of the Eating Disorder Inventory (DT: α = 0.81; BUL: α = 0.87; BD: α = 0.87) [27].

Self-Compassion Scale [5,28]. The original SCS has 26 items measuring three positive facets (i.e., self-kindness, common humanity, and mindfulness) and three negative facets (i.e., self-judgment, isolation, and overidentification). Although Neff [5] originally recommended computing a total score that combines both positive and negative items, this study calculated an SC score based solely on the positive subscales, following recent psychometric recommendations [29]. Indeed, negative items have been shown to load strongly on factors associated with general distress or self-criticism, suggesting that they do not simply represent low levels or absence of SC but may reflect broader constructs, such as general psychological distress, self-criticism, and psychopathology [30,31,32]. Moreover, in the Czech validation, separating compassionate and uncompassionate responding as separate constructs provided a better model fit than the original six-factor structure [28]. The Czech version showed good reliability for the compassionate self-responding score (α = 0.87), with higher scores indicating greater SC levels.

The Test of Self-Conscious Affect-3 (TOSCA-3S) [15,33]. The TOSCA-3S assesses four personality traits: SP, guilt proneness, externalization to others, and detachment/unconcern. However, only the SP subscale was used, given the study’s aim. Specifically, the scale presents 11 everyday scenarios, and participants select one of 4 possible responses, rating the likelihood of each on a 5-point scale (1 = not likely; 5 = very likely). Higher scores indicate a greater proneness to experience shame across various daily situations. The Czech version showed moderate reliability for the SP scale (α = 0.79) [33].

### 2.3. Data Analysis

Descriptive statistics were calculated with the Statistical Package for Social Sciences (SPSS, version 25) software. Furthermore, R Studio (version 2024.12.0 Build 467) was used to assess normality by calculating kurtosis and skewness, and to identify outliers by inspecting standardized values. Moreover, R Studio was used to perform Latent Profile Analysis (LPA), analysis of variance (ANOVA), and moderation analysis. The LPA was conducted and reported in accordance with the guidelines of Weller et al. [34] and Spurk et al. [35].

Specifically, LPA was conducted to identify latent psychological profiles within the clinical sample related to EDs, focusing on DT, BUL, and BD, using the tidyLPA package (version 1.1.0 [36]). Four latent profile models were estimated: (1) equal between-class variances and between-variable covariances set to zero; (2) varying between-class variances and between-variable covariances set to zero; (3) equal between-class variances and equal covariances; (5) varying between-class variances and varying covariances. The Expectation-Maximization (EM) algorithm was applied with default settings (i.e., a maximum of 100 iterations and a convergence threshold set so that the algorithm stops when the change in log-likelihood between successive iterations becomes smaller than ε = 1 × e^-5^). All models converged successfully without estimation warnings for the selected solution, suggesting stable results. The best model was selected based on BIC and parsimony. For each model, fit indices such as entropy, Bayesian Information Criterion (BIC), Akaike Information Criterion (AIC), and the Bootstrap Likelihood Ratio Test (BLRT) were analyzed. The model with the best fit was chosen.

Once the profiles were defined, two independent-samples ANOVAs were conducted to examine whether the profiles differed significantly in SP and SC levels. First, the Levene test was performed to verify the assumption of homogeneity of variance, and the Shapiro–Wilk and QQplot tests were used to confirm that the residuals were normally distributed. Then, Tukey multiple comparisons of means at the 95% family-wise confidence level were performed to determine whether there were statistically significant differences in SP and SC levels across the latent profiles. ANOVA and Tukey post hoc tests were performed using the car [37] package in R.

Finally, Pearson correlations and a moderation analysis were performed to verify whether SC moderated the interaction between shame and the ED scales, namely, DT, BUL, and BD. SC was dichotomized into two levels, low and high, based on the median value. The adjusted R-squared value was calculated to verify the amount of variance explained. Pearson correlations were computed using the Hmisc package [38].

## 3. Results

The standardized variables of drive for thinness (DT), bulimia (BUL), and body dissatisfaction (BD) did not exhibit minimum or maximum values greater than 3, indicating no outliers in the distributions. Skewness and kurtosis values fell within the normality range (see Table 1 for descriptive statistics).

Descriptive statistics for the psychological variables indicated that SC had a mean of 40.16 (SD = 10.19), while SP had a mean of 39.85 (SD = 7.26). Regarding EDI scales, the average score of DT was 14.15 (SD = 4.82), BUL’s mean was 4.11 (SD = 4.79), while BD had a mean of 17.96 (SD = 6.74). No missing data were found.

**Figure 1:**
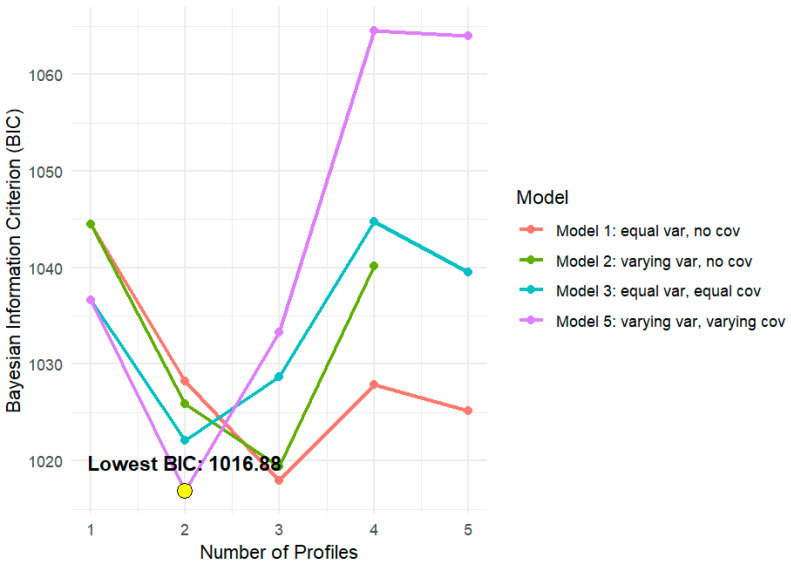
Model BIC by number of profiles. Note. The figure illustrates the behavior of the Bayesian Information Criterion (BIC) for models with an increasing number of latent profiles (ranging from 1 to 5), comparing four model specifications: Model 1 (equal variances, no covariance), Model 2 (unequal variances, no covariance), Model 3 (equal variances, equal covariances), and Model 5 (free variances and covariances). The yellow dot highlights the lowest BIC value.

Regarding LPA, the first and second models achieved the best fit with three classes, while the third and fifth models performed best with two classes (see Figure 1 and Table 2 for model comparison). 

Although slightly lower BIC values were observed for Model 5 with two classes, the differences were marginal with Model 1 and did not justify sacrificing parsimony or interpretability. Thus, Model 1 was chosen because it was more parsimonious (i.e., LogLik = −480.91; AIC = 989.83; BIC = 1017.93; CAIC = 1031.93). Specifically, Model 1 exhibited excellent class separation (H > 0.80), which was higher than that of Model 5, and balanced class sizes, with no overly small classes (N_min = 20%, N_max = 47%), which would otherwise raise concerns about overfitting or instability. BLRT was significant, indicating that the 3-class model fits significantly better than the 2-class solution (BLRT = 26.37, *p* = 0.01).

Model 1 identified three classes: (a) Low symptom group, likely less symptomatic or subclinical with low DT and BUL, moderate BD, had the low SP and high SC levels, representing possibly more adaptive or resilient group; (b) Restrictive profile with high DT and BD, minimal BUL, high SP and low SC; (c) Multi-symptomatic profile, a group with medium-high levels of DT, BUL, and BD, moderate SP, and low SC. Clinically, both the Restrictive and Multisymptomatic profiles were characterized by similarly elevated levels of DT and BD. However, they differed markedly in BUL. Indeed, the Restrictive profile showed very low levels of bulimia. In contrast, the Multisymptomatic profile was defined by clearly elevated bulimic symptoms, indicating the co-occurrence of binge–purge behaviors.

**Figure 2:**
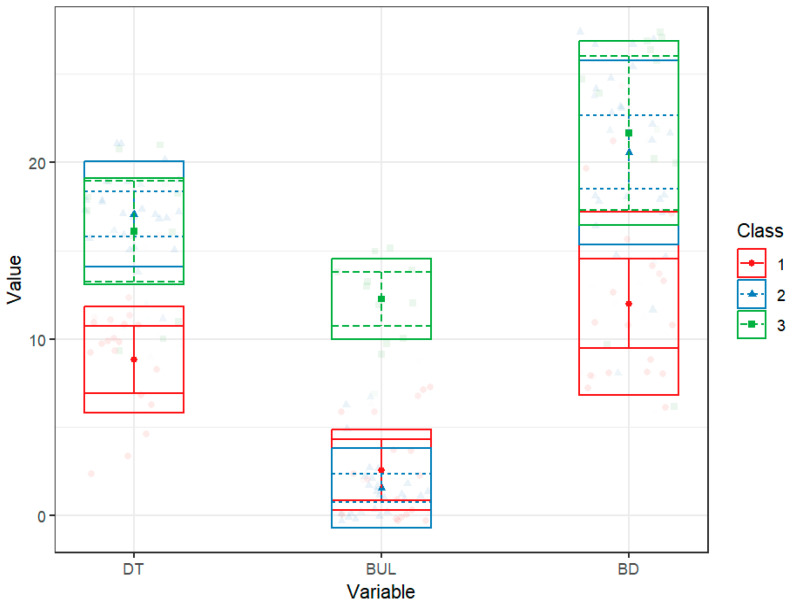
Distribution of psychopathological scores in the three latent profiles. Note. The figure illustrates the distribution differences in individual scores between the latent profiles in Drive for Thinness (DT), Bulimia (BUL), and Body Dissatisfaction (BD) scores within the three identified classes: Class 1 (red), Class 2 (blue), and Class 3 (green). The graph displays the estimated means of the scores for each profile (squares in the center of the box), along with 95% confidence intervals (vertical lines extending from the center square) and standard deviations above and below the mean (horizontal lines above and below the center square). The colored dots represent the individual observed data for each class.

In the chosen three-profile solution, 18 participants (32.7%) were assigned to Profile 1, 26 (47.3%) to Profile 2, and 11 (20.0%) to Profile 3. Average posterior probabilities (AvePP) for the most likely class membership were 0.96, 0.96, and 0.99 for Profiles 1, 2, and 3, respectively, demonstrating high classification accuracy and clear separation between profiles despite the modest sample size. Table 3 displays the means of the EDI scales for each profile, along with the numerosity, proportions, and AvePP values of each class, and Figure 2 shows the box plots for these profiles.

**Figure 3:**
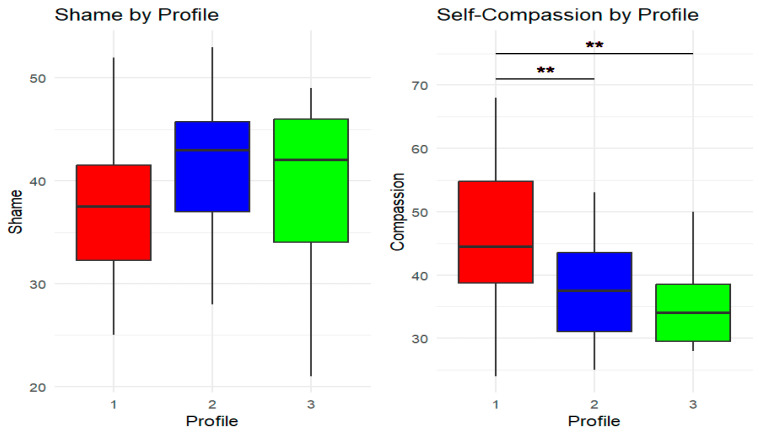
Post hoc comparison of shame-proneness and self-compassion scores across latent profiles. Note. The figure illustrates the distribution of shame-proneness (**left**) and self-compassion (SC, (**right**)) scores across the three identified latent profiles. Each boxplot represents the median (thick horizontal line), the interquartile range (box), and the minimum and maximum values within 1.5 times the interquartile range (vertical lines, or “whiskers”). The colors represent the latent profiles (Profile 1 = red, Profile 2 = blue, Profile 3 = green). The double red asterisks (**) indicate significant differences in SC scores between profiles. Profile 1 shows significantly higher SC levels than Profiles 2 and 3. Shame-proneness levels, however, tend to increase in the more symptomatic profiles, but the differences are not statistically significant.

Levene’s test was not significant for SP (F (2, 52) = 0.62, *p* = 0.54) and SC (F (2, 52) = 2.04, *p* = 0.14), indicating homogeneity of variance. Similarly, Shapiro–Wilk’s test was not significant for SP (W = 0.986, *p* = 0.77) and SC (W = 0.986, *p* = 0.779), suggesting that the residuals follow a normal distribution. Q-Q Plots confirmed visual normality for both models. ANOVA revealed no statistically significant differences in SP among the three profiles (F (2, 52) = 1.73, *p* = 0.188, η^2^ = 0.06). Conversely, the model for SC was significant (F (2, 52) = 6.85, *p* = 0.002, η^2^ = 0.21). Specifically, Tukey’s post hoc tests showed that group one, with the lowest symptom level, showed statistically higher SC than the second (Mdiff = −9.30, *p* = 0.005) and third groups (Mdiff = −10.81, *p* = 0.009), characterized by restrictive and mixed symptomatology, respectively (see Figure 3 for post hoc comparisons).

Pearson correlations indicated that SC was negatively correlated with all EDI subscales. Specifically, an increase in SC was linked to lower levels of DT (r = −0.46, *p* < 0.001), BUL (r = −0.20, *p* = 0.153), BD (r = −0.41, *p* = 0.002), and SP (r = −0.48, *p* < 0.001), suggesting that as SC increased, eating symptoms and experiences of shame tended to decrease. In contrast, SP was positively correlated with the EDI scales; only the correlation with DT was significant (r = 0.31, *p* = 0.020), whereas those with BUL (r = 0.04, *p* = 0.796) and BD (r = 0.12, *p* = 0.382) were not.

**Figure 4:**
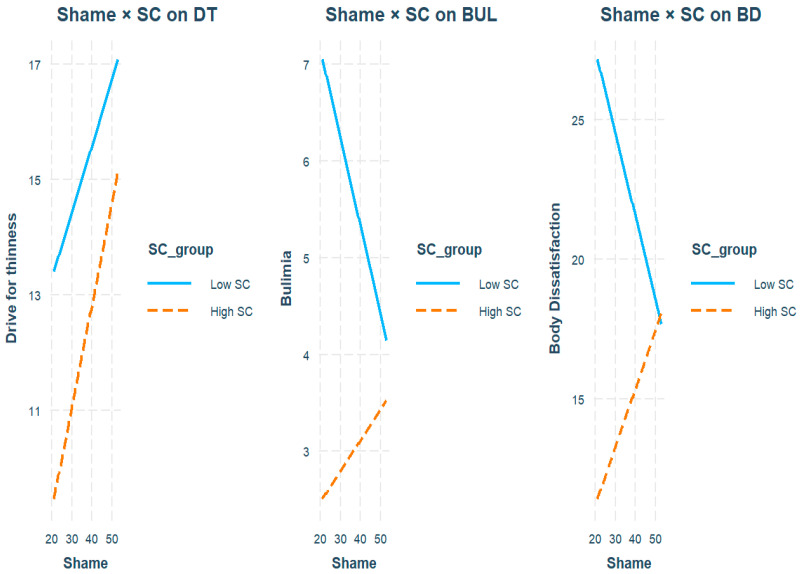
Moderation analysis. Note. The figure illustrates the interactions between shame-proneness and self-compassion (SC) in predicting Drive for Thinness (DT), Bulimia (BUL), and Body Dissatisfaction (BD). The graphs display the regression lines, categorized by SC level (low = solid blue; high = dashed orange). For BD, the interaction is significant: at low levels of shame, high SC is associated with markedly lower BD than low SC, but this difference decreases as shame increases, with the two lines converging at higher levels of shame. This pattern suggests that SC attenuates the impact of shame on BD when shame is relatively low to moderate, but this buffering effect weakens at high levels of shame. For DT and BUL, the interactions are not significant.

Finally, the moderation analysis revealed no significant effects of SP, SC, or their interaction on DT (see Table 4 and Figure 4). Nevertheless, the model remained statistically significant (F (3, 51) = 3.61, *p* = 0.019), accounting for 18% of the variance. In the case of BUL, no predictor achieved statistical significance, and the overall model was statistically nonsignificant (F (3, 51) = 1.05, *p* = 0.380; R^2^ = 0.06). In contrast, the model for BD showed significant effects for the high-SC group (b = –26.30, SE = 9.53, t = –2.76, *p* = 0.008) and for the interaction between SP and SC (b = 0.50, SE = 0.23, t = 2.15, *p* = 0.036). The model explained 27% of the variance and was significant (F (3, 51) = 6.33, *p* = 0.001), suggesting that SC moderated the relationship between shame and BD. Inspection of the interaction pattern indicated that, at low levels of SP, women with high SC reported substantially lower BD than those with low SC. In contrast, this difference progressively diminished as SP increased, with BD converging to similarly high levels in both groups (see Figure 4). Thus, higher SC was associated with lower BD, particularly when shame was relatively low to moderate, but this protective effect weakened at higher levels of shame.

Note. This table illustrates how shame-proneness, low SC, and high SC predict the EDI scales (i.e., DT, BUL, and BD), and how different levels of SC moderate the relationship between shame and the EDI scales. Low SC levels, that is, those below the median, represent the baseline, or intercept. SC = Self-Compassion; SP = Shame-proneness; DT = Drive for Thinness; BUL = Bulimia; BD = Body Dissatisfaction.

## 4. Discussion

This exploratory study aimed to identify distinct symptomatic profiles within the ED female sample and determine whether levels of SC and SP varied across classes. According to LPA, Model 1 was more parsimonious and effectively differentiated participants based on their symptom profiles. Three profiles of ED symptomatology emerged. Specifically, the low-symptom group, characterized by low DT and BUL with moderate BD; the restrictive profile, characterized by high DT and BD but minimal BUL; and the multi-symptomatic profile, which showed medium to high levels of DT, BUL, and BD. These profiles align with prior research, including studies using LPA [39,40]. Additionally, the Restrictive and Multisymptomatic profiles shared a common core marked by high DT and prominent BD, while differing mainly in the presence or absence of significant BUL. This pattern might indicate a severity continuum in bulimic symptoms, along with a shared core of restrictive and weight–shape concerns, rather than representing entirely separate latent phenotypes. Consistent with this view, longitudinal studies have shown frequent diagnostic crossover from restrictive to binge–purge forms of AN, and between AN and BN, implying that the appearance of bulimic symptoms often reflects a change in severity and symptom profile within a typical psychopathological spectrum rather than a complete shift to a different disorder (e.g., [24]).

Furthermore, while shame levels did not differ between classes, the subclinical or less symptomatic group showed higher levels of SC than the restrictive and multisymptomatic groups. This significant role of SC aligns with the literature, as higher SC levels are associated with the less symptomatic group [41]. Consequently, SC is confirmed as a likely protective factor against EDs [7]. Conversely, the lack of significant differences in SP between profiles contrasts with the existing literature. For example, O’Loghlen et al. [19] found that symptomatically complex profiles showed the highest levels of shame, while Cavalera et al. [18] found that SP levels were higher in ED patients than in subthreshold people or controls. However, O’Loghlen et al. [19] used LPA to distinguish between different binge-eating profiles, and Cavalera et al. [18] also recruited healthy individuals. In contrast, this study involved only patients experiencing AN and BN. Importantly, given the modest sample size and the small number of participants in each profile, especially in the smallest class, the non-significant differences in SP should be interpreted with caution, as the study may have been underpowered to detect small or subtle between-profile differences in shame. From a conceptual standpoint, the current pattern is consistent with the idea that SP may function as an underlying vulnerability factor that tends to show relatively similar levels across different ED symptom profiles. Still, larger, adequately powered studies are needed to test whether SP functions as a more definitive transdiagnostic vulnerability factor.

Moreover, SC correlated negatively with shame DT and BUL, such that as SC levels increase, symptoms decrease, in line with other studies [7,8,10]. Conversely, SP correlated positively only with BD, a result partially confirmed, given that shame is also linked with other ED symptoms in other studies [16,17]. Additionally, moderation analysis revealed that SC attenuated the association between shame and BD (i.e., women with high SC reported markedly lower BD than those with low SC at low to moderate levels of shame). In contrast, this buffering effect weakened as shame increased and did not occur for BUL and DT. These findings are confirmed partially in the literature. For example, SC moderates the effect of shame memories on memories of ED symptom severity [11] and diminishes the size of the association between self-disgust and DT [12]. Consequently, although SC’s moderation is confirmed for the shame–BD relationship, the lack of association with other constructs, such as DT, is not in line with the literature. Perhaps the heterogeneous operationalization of SP or SC may be responsible for producing discordant results. For example, this study investigated SP, whereas others have examined a more specific form of shame, focusing on the body or external aspects [11,12]. Therefore, SC may exert a broad protective role specifically for BD, particularly when shame is not extreme, diminishing the impact of shame on BD in females with ED symptoms [23,42]. In contrast, similar levels of shame across symptom profiles are consistent with the hypothesis that SP functions as an underlying vulnerability factor for females with ED [24,25]. Nevertheless, the modest sample size may have limited the statistical power of the moderation models, particularly for detecting interaction effects, so that non-significant interactions should be interpreted with caution. Thus, the apparent specificity of the SC’s moderation effect on the SP-BD relationship may at least partly reflect insufficient power rather than a genuine absence of moderation for the other symptom dimensions.

From a clinical perspective, these results support the implementation of interventions that explicitly integrate self-compassion and mindfulness-based components, such as practices aimed at cultivating present-moment awareness of shame and body-related distress, held with curiosity and kindness rather than avoidance or self-attack [14]. Such mindfulness-informed SC interventions may be beneficial for restrictive and multi-symptomatic profiles, helping patients to decenter from rigid appearance ideals, soften perfectionistic self-criticism, and reframe their suffering within a shared human experience [5,14]. Consequently, therapeutic interventions for EDs should consider that groups of patients with different symptoms could benefit from different therapies. For example, the restrictive group would benefit from learning to be more compassionate with themselves through self-acceptance and a decrease in perfectionistic ideals, thereby reducing the shame associated with not adhering to a standard that drives body thinness. Interestingly, SC-based interventions are effective in reducing ED symptom levels [6,7,8]; however, no studies have yet differentiated the power of the intervention effect among different clinical ED subtypes.

### 4.1. Limitations

Although the study moves beyond traditional categorical diagnoses, allowing for a more dimensional and heterogeneous view of the clinical manifestations that contribute to a deeper understanding of the underlying mechanisms of EDs, it also has several limitations. First, the sample size was modest (*n* = 55), and the smallest latent profile included only 11 participants (20% of the sample). Although the classification indices were satisfactory (high entropy, high AvePP, and good separation between profiles), the stability and generalizability of the identified profiles remain uncertain. For this reason, the LPA results should be considered preliminary and exploratory, and replication in larger and more diverse clinical samples is needed. Moreover, a small sample size might have prevented the detection of the SP’s effect in the ANOVA and moderation analysis. Second, including only female participants limits the generalizability of the findings to males, who might exhibit different patterns of SC, SP, and ED symptoms. Third, BMI and illness duration were not systematically recorded across all participants. Although BMI was assessed as part of the diagnostic process for AN, the exact values were not documented in a standardized format for research purposes, preventing their inclusion in the analyses. Illness duration was not consistently available. This limits our ability to evaluate how somatic severity and chronicity may have influenced the observed associations. Fourth, the cross-sectional design prevents any conclusions about causality or the direction of relationships between SC, SP, and ED symptoms. Fifth, SC was operationalized using only the compassionate self-responding items of the SCS. Although this approach is supported by recent psychometric literature, it does not capture uncompassionate self-responding (self-judgment, isolation, over-identification), limiting direct comparability with studies using the original total SCS score. Finally, since the study was conducted in the Czech Republic, cultural factors could influence both SC and shame experiences, which limits the ability to generalize the findings to other cultural contexts. Indeed, the Czech sociocultural context is increasingly exposed to Westernized beauty and thin-ideal standards, which may contribute to elevated levels of DT and BD in women with ED. However, we did not directly assess sociocultural influences in this study, so any interpretation regarding cultural effects remains speculative and should be regarded as a limitation when extrapolating these findings beyond the Czech context.

### 4.2. Future Directions

The present findings should be viewed as exploratory and need to be replicated. Therefore, future studies should include larger samples from diverse cultural backgrounds to enhance the generalizability and stability of LPA solutions for complex models. Additionally, longitudinal designs that utilize transient LPA or dynamic networks could help clarify the causal and temporal relationships between SC, SP, and ED symptoms. Experimental studies should investigate whether SC is a change mechanism or process, examining whether individuals who receive SC treatment show greater improvement than those who do not. Finally, future work may also benefit from distinguishing between different types of shame (e.g., body shame, external shame, internal shame) and modeling both components of SC (i.e., positive and negative) to investigate which aspects are most closely linked to ED symptoms.

## 5. Conclusions

To conclude, this study emphasizes the diversity of ED symptomatology by identifying distinct latent profiles and examining the relationship between SC and SP within these groups. Although SP levels did not significantly vary across these profiles, SC acted as a protective factor, with lower levels found in more symptomatic groups and moderating the link between shame and BD. These results support the incorporation of SC-focused interventions into clinical practice to reduce BD and potentially mitigate the negative impact of shame. Future longitudinal and intervention studies are needed to better understand these mechanisms and evaluate the effectiveness of targeting SC across different clinical populations.

## Figures and Tables

**Table 1:** Sociodemographic and clinical characteristics.

Variable	Category	N (%)	M (SD)	Skewness	Kurtosis
Diagnosis	F50.0 (Anorexia nervosa)	30 (54.5)			
	F50.2 (Bulimia nervosa)	25 (45.5)			
Sex	Female	55 (100.0)			
Marital status	Single	48 (87.3)			
	Married	5 (9.1)			
	Divorced	2 (3.6)			
Children	No	45 (81.8)			
	Yes	10 (18.2)			
Education	Primary education (1st–9th grade)	3 (5.5)			
	Secondary education (apprenticeship)	16 (29.1)			
	Secondary education (maturity exam)	13 (23.6)			
	Tertiary education	23 (41.8)			
Age		55	26.18 (9.10)	1.60	2.52
SC		55	40.16 (10.19)	0.79	0.57
SP		55	39.85 (7.26)	−0.38	−0.37
DT		55	14.15 (4.82)	−0.60	−0.44
BUL		55	4.11 (4.79)	1.07	−0.18
BD		55	17.96 (6.74)	−0.25	−1.22

Note. This table presents descriptive statistics for demographic and clinical information; SC = Self-Compassion; SP = Shame-proneness; DT = Drive for Thinness; BUL = Bulimia; BD = Body Dissatisfaction.

**Table 2:** LPA model selection.

Model	Classes	LogLik	AIC	BIC	H	Pmin	Pmax	Nmin	Nmax	BLRT *p*
Model 1	3	−480.91	989.83	1017.93	0.90	0.95	0.97	0.20	0.47	0.01
Model 2	3	−469.60	979.21	1019.36	0.90	0.95	1.00	0.13	0.53	0.02
Model 3	2	−484.98	995.96	1022.05	0.94	0.94	0.99	0.20	0.80	0.01
Model 5	2	−470.37	978.74	1016.88	0.87	0.95	0.97	0.40	0.60	0.01

Note. LogLik = log-likelihood; AIC = Akaike Information Criterion; BIC = Bayesian Information Criterion; H = entropy; Pmin/Pmax = minimum/maximum class probability; Nmin/Nmax = smallest/largest class size (%); BLRT *p* = bootstrap likelihood ratio test *p*-value.

**Table 3:** Latent profile characteristics.

Profile	DT	BUL	BD	SP	SC	N	% of Sample	AvePP
1—Low symptom	8.67	2.67	11.90	37.50	46.70	18	32.70	0.96
2—Restrictive	17.20	1.58	20.60	41.60	37.40	26	47.30	0.96
3—Multisymptomatic	16.00	12.50	21.60	39.60	35.90	11	20.00	0.99

Note. This table displays the means of the EDI scales for each profile, along with the numerosity, proportions, and average posterior probabilities (AvePP) values of each class. DT = Drive for Thinness; BUL = Bulimia; BD = Body Dissatisfaction; SP = shame-proneness; SC = self-compassion. Percentages may not sum to 100 due to rounding.

**Table 4:** Moderation analysis.

Outcome	Predictor	B	SE	t	*p*	R^2^/Adj. R^2^	F (df)	*p* (Model)	Residual SE
DT	SC_Low (Intercept)	10.98	5.64	1.95	0.057	0.18/0.13	3.61 (3, 51)	0.019	4.51
	SP	0.12	0.13	0.87	0.389				
	SC_High	−5.23	7.26	−0.72	0.474				
	SP × SC_High	0.06	0.18	0.35	0.731				
BUL	SC_Low (Intercept)	8.96	5.98	1.50	0.140	0.06/0.00	1.05 (3, 51)	0.380	4.78
	SP	−0.09	0.14	−0.65	0.521				
	SC_High	−7.12	7.70	−0.93	0.360				
	SP × SC_High	0.12	0.19	0.65	0.520				
BD	SC_Low (Intercept)	33.38	7.40	4.51	0.000	0.27/0.23	6.33 (3, 51)	0.001	5.92
	SP	−0.30	0.17	−1.70	0.095				
	SC_High	−26.30	9.53	−2.76	0.008				
	SP × SC_High	0.50	0.23	2.15	0.036				

## Data Availability

The raw data supporting the conclusions of this article will be made available by the authors on request.

## Abbreviations

The following abbreviations are used in this manuscript:

SC	Self-Compassion
SP	Shame-Proneness
BD	Body Dissatisfaction
EDs	Eating Disorders
AN	Anorexia Nervosa
BN	Bulimia Nervosa
DT	Drive for Thinness
BUL	Bulimia
LPA	Latent Profile Analysis
